# The Relationship Between Adjustment and Mental Health of Chinese Freshmen: The Mediating Effect of Security and the Moderating Effect of Gender

**DOI:** 10.3389/fpubh.2022.916329

**Published:** 2022-05-23

**Authors:** Liu Cao

**Affiliations:** School of Physical Education, Xuzhou University of Technology, Xuzhou, China

**Keywords:** adjustment, mental health, security, moderating effect, mediating effect

## Abstract

During the COVID-19 pandemic, many universities are confronted with campus lockdown or even school closures to reduce the risk of infection. However, these measures pose a threat to the mental health of adolescents. In particular, freshmen who have just entered the university campus may suffer from more serious mental health risks. In this study, 1,818 freshmen were analyzed by using the Chinese College Student Adjustment Scale (CCSAS), Sense of Security Questionnaire (SQ), and Symptom Check List 90 (SCL-90) of the qualitative method. The results showed that adjustment had an impact on mental health. Firstly, there was a significant negative association between adjustment and mental health. Secondly, adjustment had a significant predictive effect on mental health. Finally, a sense of security and gender affected the relationship between adjustment and mental health. Collectively, adjustment, sense of security, and gender exerted impacts on the mental health of freshmen, suggesting that we should create a warm and harmonious campus environment for students and conduct targeted education for male and female students.

## Introduction

Data from recent years indicate that mental health issues have constituted significant burdens affecting a considerable number of people worldwide. Particularly, in the context of the novel coronavirus disease 2019 (COVID-19) pandemic, the mental health and wellbeing of people are experiencing critical challenges, leading to a global mental health crisis (1). A variety of COVID-19 pandemic-triggered mental health problems has been reported in different groups in society, such as stress, anxiety, depression, and sleep disorders (2–4). What's more, negative emotions and related problems in daily life, work, or study continue to rise with the spread of the epidemic. To curb the spread of the virus, many countries have to practice different social distancing measures, such as a lockdown of the campus and a shift to distance or online learning (5). Sifat et al. (6) have noted that adolescents are more susceptible to mental health issues during the lockdown. University freshmen are a special population enduring a critical period of exploring the campus and socializing with peers. However, the school closure brought by the COVID-19 pandemic forces them to adapt to online learning and exams and reduce interactions with peers (7), which is unfavorable to the mental health of adolescents and psychosocial development (8). Therefore, the research on the relationship between adjustment and mental health has received increasing academic attention. The present study aims to investigate the relationship between adjustment and mental health among Chinese university freshmen, hoping to provide a basis for the school and society to give accurate psychological aid to university students.

Adjustment is primarily the response to psychosocial stressors or multiple stressors, which is accepted as an important response indicator of mental health. According to the diagnostic criteria provided in the Diagnostic and Statistical Manual of Mental Disorders-5th edition (DSM-5), individuals with adjustment disorders are accompanied by significant emotional and behavioral symptoms (9). Evidently, there is a high correlation between adjustment and mental health. Accumulating studies have elucidated the significant negative correlation between mental health and adjustment in college students (10–12). Such negative correlation has also been validated in other groups. For instance, the study by Melero et al. (13) has suggested that adults who are adopted during childhood tend to develop more adjustment problems and present a higher incidence of depression, anxiety, and personality and behavioral disorders than the general population. A study of young adults who have experienced stress events demonstrates that higher levels of mental health are correlated with fewer symptoms of adjustment disorders (14). A literature review has revealed a negative relationship between adjustment and mental health, but there are few studies on freshmen. Herein, this study proposes hypothesis 1: adjustment exerts a significant effect on the mental health of freshmen.

A sense of security is considered to be an important influencing factor in regulating the relationship between adjustment and mental health (15). A sense of security is a sense of control over life, which reflects an individual's ability to deal with life problems and the belief that an individual is liked and accepted by himself and others (16). Maslow believes that people with a low sense of security tend to remain alert, feel isolated, and often exhibit tension and introversion (17). Emotional Security Theory states that maintaining and preserving a sense of security is the most basic task of an individual (18). When a negative interpersonal environment (e.g., interpersonal blocking) undermines personal security, individuals take various measures to defuse or avoid these threats to maintain their security (18, 19). Accordingly, Jia et al. (20) have noted that a sense of security is a key mediator between adjustment (e.g., interpersonal relationships) and problem behaviors. Geng et al. (21) have also found that a sense of security is a mediator between interpersonal sensitivity (e.g., shyness) and depressive symptoms. Moreover, the mediating role of security between peer relationships and internet addiction has been documented (20). The generation of a sense of security is a complex process, in which the environment is a pivotal influencing factor that has an impact on the individual's physiology and psychology. Among students, those with a higher sense of security and belonging have a higher level of mental health (22). Therefore, based on the Emotional Security Theory, the study proposes hypothesis 2: a sense of security plays a mediating role in the relationship between adjustment and mental health.

**Figure 1:**
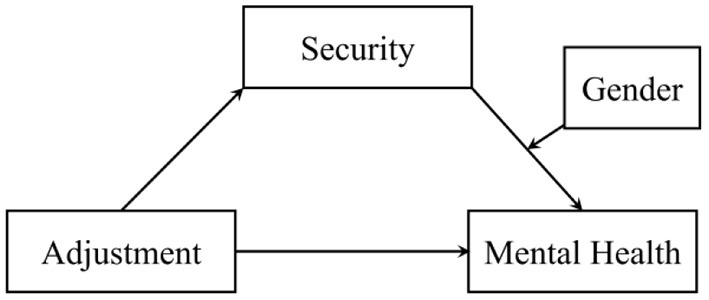
Relationship between adjustment and mental health: the effect of security and gender.

Age and gender are significant psychological and physiological variables (23). During the COVID-19 pandemic, adolescents suffer more social isolation and family stress and experience more depression and anxiety, and furthermore, the severity of mental health problems in women is much higher than that in men (4). Accordingly, this study proposes hypothesis 3: gender moderates the relationship between a sense of security and mental health. Based on the hypotheses, this study proposes a conceptual model map, as shown in Figure 1.

## Materials and Methods

### Participants

The freshmen from six universities in Jiangsu Province were investigated by random sampling. This study was approved by the college ethics committee. The subjects were informed of the purpose of the survey and informed consent was obtained before the survey. A total of 2,000 questionnaires were distributed and 1,873 were received, with a questionnaire response rate of 93.7%; the final number of valid subjects was 1,818, with an effective rate of 97.1%. Among the valid subjects, 816 (44.9%) were male and 1,002 (55.1%) were female, ranging from 17 to 24 years old, with an average age of 19.47 ± 1.18 years old.

### Instruments

Chinese College Student Adjustment Scale (CCSAS), Sense of Security Questionnaire (SQ), and Symptom Check List 90 (SCL-90) were adopted.

(a) Chinese College Student Adjustment Scale (CCSAS). Fang et al. (24) prepared the scale with 60 items, including 7 dimensions of interpersonal adjustment, academic adjustment, campus life adjustment, career choice adjustment, emotional adjustment, self-adjustment, and satisfaction. A 5-point scale was used, ranging from 1 point (disagree) to 5 points (agree), but some entries were reverse-scored. A higher score was indicative of a higher level of adjustment. This scale had good reliability and validity, with the Cronbach's alpha ranging from 0.67 to 0.82 for the 7 dimensions and 0.93 for the total scale.
(b) Sense of Security Questionnaire (SQ). Cong and An (25) formulated the scale with 16 items, which were divided into 2 factors: interpersonal security and certainty in control. The scale was scored on a 5-point scale from 1 point (very consistent) to 5 points (very inconsistent), with a higher score indicating a higher sense of security. The Cronbach's alpha was 0.75 for interpersonal security, 0.72 for certainty in control, and 0.80 for the total scale.
(c) Symptom Check List 90 (SCL-90). The scale was prepared by Derogatis and revised by Liu and Zhang (26). The scale contained a total of 90 items, including 9 factors such as somatization, obsessive-compulsive, interpersonal sensitivity, depression, anxiety, hostility, phobia anxiety, paranoid ideation, and psychoticism, and 1 other factor, and these 10 factors constituted the total mental health score. A 5-point scale was used, ranging from 1 point (none) to 5 points (severe). Higher factor scores and total scores were associated with more prominent psychological problems. The Cronbach's alpha of the 10 factors for secondary school students ranged from 0.70 to 0.88.

### Procedures

The test was conducted by team members who had received systematically training and obtained a qualification in measurement before the test. The test was conducted as a group of a class, and the time limit was about 30 min. All questionnaires were distributed and recovered on the spot. The recovered questionnaires were checked by a team member and then a database was created. The data were analyzed and processed using SPSS27.0 and the Macro Program Process developed by Hayes. The gender differences in adjustment, sense of security, and mental health were analyzed using the independent sample *t*-test, and the correlation between adjustment, sense of security, and mental health was analyzed using Pearson product-moment correlation coefficient. The mediating effect of security and the moderating effect of gender were analyzed using the Macro Program Process developed by Hayes. The bootstrap method was used, with a sample size of 5,000 and a confidence interval of 95%.

## Results

### Difference Comparison

As shown in Table 1, there were significant differences in the scores of SCL-90, CCSAS, and SQ scales between male and female students. Specifically, female students got higher scores than male students in SCL-90, and male students got higher scores than female students in CCSAS and SQ. According to the difference comparison results and previous analysis, it could be concluded that gender played a moderating role in the relationship between SQ and SCL-90.

**Table 1:** Comparison of the differences in SCL-90, CCSAS, and SQ among freshmen of different genders (*n* = 1,818).

**Variables**	**Female**	**Male**	***t***
SCL-90	1.70 ± 0.43	1.57 ± 0.40	6.67***
CCSAS	3.31 ± 0.55	3.39 ± 0.56	−3.17**
SQ	3.55 ± 0.67	3.75 ± 0.68	−6.33***

** *p < 0.01*;

*** *p < 0.001*.

### Descriptive Statistics and Correlation Analysis

The correlation analysis revealed (Table 2) that CCSAS, SQ, and SCL-90 were significantly correlated with gender. CCSAS, SQ, and SCL-90 showed a significant negative correlation, while CCSAS and SQ showed a significant positive correlation.

**Table 2:** Descriptive statistics and correlation analysis of the variables.

	**M**	**SD**	**SCL-90**	**CCSAS**	**SQ**	**Gender**
SCL-90	1.64	0.42	1			
CCSAS	3.35	0.56	−0.49**	1		
SQ	3.64	0.68	−0.60**	0.67**	1	
Gender	–	–	0.07**	0.15**	−0.16**	1

** *p < 0.01*.

### Moderating and Mediating Effects Analyses

According to the test method of the structural equation model and the results of correlation analysis, the conditions for testing moderated mediation model were satisfied, so the mediating role of SQ and the moderating role of gender were examined in the relationship between CCSAS and SCL-90. CCSAS, SQ, and SCL-90 were standardized before the analysis, with female = 0 and male = 1, and then, the effect test was performed.

The analysis found (Table 3) that CCSAS, SQ, and gender had a significant main effect on SCL-90 [effect = −0.17, *p* < 0.001, 95% CI (−0.22 to −0.12); effect = −0.52, *p* < 0.001, 95% CI (−0.58 to −0.46); effect = −0.15, *p* < 0.01, 95% CI (−0.22 to −0.07)]. The main effect of CCSAS on SQ was also significant [effect = 0.67, *p* < 0.001, 95% CI (0.63–0.70)]. SQ interacting with gender showed a significant predictive effect on SCL-90 [effect = 0.09, *p* < 0.05, 95% CI (0.02–0.16)]. The main effect and interaction effect analyses indicated that the mediating role of SQ and the moderating role of gender could be further analyzed.

**Table 3:** Relationship between CCSAS and SCL-90 in freshmen: the model of SQ and gender.

**Predictor**	**Outcome variable**	**R**	**R^**2**^**	**F**	**β**	***t***	**LLCI**	**ULCI**
**variable**				
**Equation 1**
CCSAS	SQ	0.67	0.45	1,455.58***	0.67	38.15***	0.63	0.70
**Equation 2**
CCSAS	SCL-90	0.62	0.38	281.04***	−0.17	−6.13***	−0.22	−0.12
SQ					−0.52	−16.99***	−0.58	−0.46
Gender					−0.15	−3.92**	−0.22	−0.07
SQ × gender					0.09	2.41*	0.02	0.16

* *p < 0.05*;

** *p < 0.01*;

*** *p < 0.001*.

**Figure 2:**
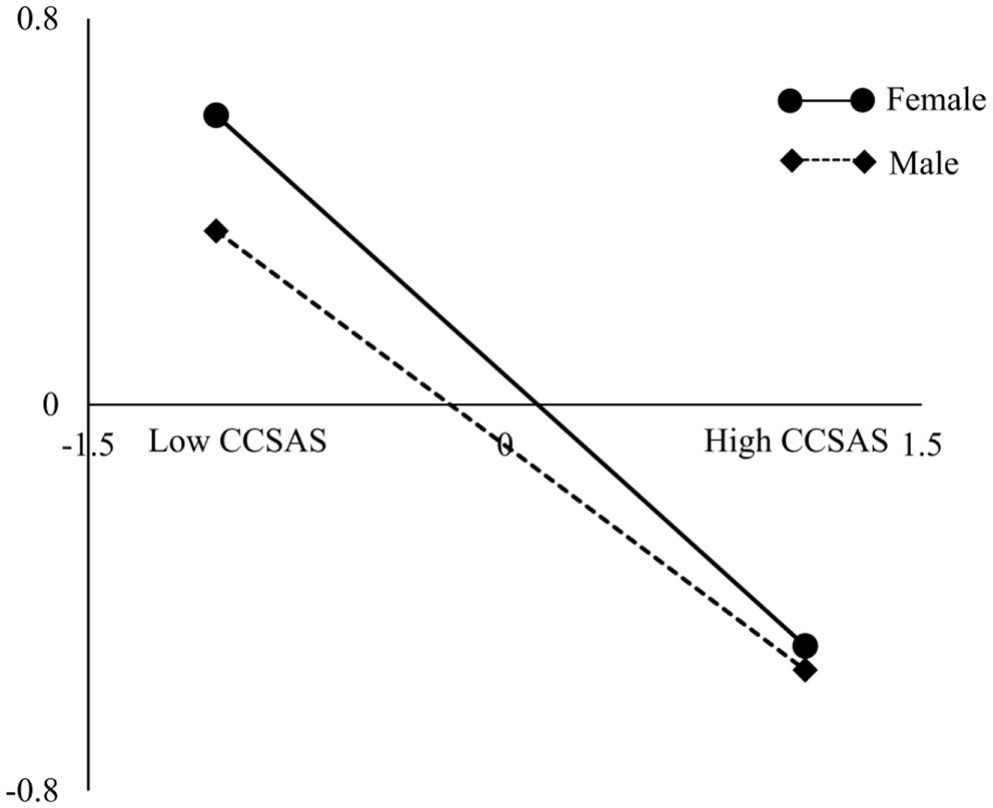
Relationship between SQ and SCL-90: the effect of gender.

The analysis revealed the moderating effect of gender in the relationship between SQ and SCL-90, wherein in the male group, the effect value of SQ on SCL-90 was significant [effect = −0.52, *P* < 0.001, 95% CI (−0.58 to −0.46)] and in the female group, the effect value of SQ on SCL-90 was also significant [effect = −0.43, *P* < 0.001, 95% CI (−0.49 to −0.37)]. The slope analysis showed (Figure 2) that the level of SCL-90 was elevated with the increase of SQ, but the change in the female group was more obvious than that in the male group.

Further mediating effect analysis (Table 4) demonstrated that the indirect effect of CCSAS → SQ → SCL-90 was −0.35 for the female group, accounting for 67.3% of the total effect and the indirect effect of CCSAS → SQ → SCL-90 was −0.29 for the male group, accounting for 63% of the total effect. It was indicated that a sense of security was a vital mediating variable in the relationship between adjustment and mental health.

**Table 4:** Relationship between CCSAS and SCL-90 of freshmen: the effect of SQ and gender.

	**Female**		**Male**	
	**Effect**	**Ratio of indirect to total effect**	**Effect**	**Ratio of indirect to total effect**
Indirect effect	−0.35	67.3%	−0.29	63%
Direct effect	−0.17	32.7%	−0.17	37%
Total effect	−0.52	–	−0.46	–

## Discussion

The study revealed the mechanism of adjustment in mental health. Security played a mediating role in the relationship between adjustment and mental health and such mediating effect was moderated by gender. The main contribution of this study is to analyze the relationship between adjustment and mental health, especially the role of security, and also provide further evidence for understanding the role of gender in the above relationships.

It was found that adjustment was positively correlated with a sense of security, and security was negatively correlated with mental health. The sense of security refers to an individual's sense of anticipation of possible physical and psychological risks and a sense of power/powerlessness in difficulty, mainly in the form of interpersonal security and certainty in control. Adjustment, on the other hand, is a reaction or state in the face of a stressor. When facing a new environment, individuals will make predictions and find ways to make active adjustments. If individuals can gain a sense of security during adjustment, they will continue to promote adjustment and maintain healthy psychology; if not, they will have difficulties in adjustment, leading to mental health problems. Therefore, security is a major variable linking adjustment and mental health. Consistently, Geng et al. (21) have validated Emotional Security Theory and Davies et al. (19) have stated that maladjustment is related to interpersonal stress and it is the personal security crisis caused by maladjustment and stress that leads to mental health problems. Especially university freshmen are facing interpersonal and academic adjustments, but the blockade brought by the COVID-19 pandemic triggers interpersonal and academic stress, which can lead to mental health problems such as depression and anxiety. In this process, the role of a sense of security becomes prominent. If the school gives students a sense of belonging and security and guides them to adjust positively, the emergence of mental health symptoms will be retarded (22).

More importantly, this study identified the role of gender in the relationship between mental health, adjustment, and security. Gender moderated the relationship between security and mental health, thus exerting an important influence on the relationship between the three, with greater changes in the female group in comparison to the male group. A survey has shown that the prevalence of any mood disorder in Chinese women is higher than that in men (27). Lu et al. (28) has also observed a higher prevalence of depressive disorders in Chinese women than men. Similarly, this study explored the role of gender in the relationship between CCSAS, SQ, and SCL-90 and also found greater changes in females. In the context of Chinese culture, the female is usually defined as tenderness, kindness, and introversion, among which sensitivity is a major trait. Male, on the other hand, are usually characterized as masculinity, self-confidence, and a sense of responsibility, among which perseverance is a major trait. The different male and female traits have different effects on the adjustment, sense of security, and mental health of students. In addition, in Chinese society and culture, men have more social and competitive opportunities than women, which also leads to gender differences. It is evident that gender continues to be an important factor in life change in China.

## Conclusion

This study explores the relationship between adjustment and mental health and determines the multiple effects of security and gender. The relationship between adjustment and mental health can be mediated through a sense of security, and gender moderates the relationship between security and mental health. The findings of this study provide a direction for the current mental health education.

Based on the above findings, we call on universities and relevant responsible departments to make full use of social networking platforms to guide freshmen to communicate and also create a healthy and positive social networking environment to cope with the obstacles brought by school closures. Teaching activities and social communication activities should be actively carried out within the limited space to enable freshmen to integrate into the new environment as soon as possible. On the premise of ensuring safety, universities should actively organize various campus activities to enrich students' after-school life and help them cope with the boring and isolated campus life. In addition, our results suggest that future school mental health education should be committed to creating an environment with a sense of security and belonging, and provide different educational activities for male and female students.

## Data Availability Statement

The original contributions presented in the study are included in the article/supplementary material, further inquiries can be directed to the corresponding author/s.

## Ethics Statement

The studies involving human participants were reviewed and approved by Ethics Committee of School of Physical Education, Xuzhou Institute of Technology. The patients/participants provided their written informed consent to participate in this study.

## Author Contributions

LC: conception and design, collection and assembly of data, data analysis and interpretation, manuscript writing, and final approval of manuscript.

## Funding

This work was supported in part by the Xuzhou Science and Technology Plan Project under Grant KC21303, Jiangsu Industry University Research Cooperation Project under Grant BY2021159, and the sixth 333 project of Jiangsu Province.

## Conflict of Interest

The author declares that the research was conducted in the absence of any commercial or financial relationships that could be construed as a potential conflict of interest.

## Publisher's Note

All claims expressed in this article are solely those of the authors and do not necessarily represent those of their affiliated organizations, or those of the publisher, the editors and the reviewers. Any product that may be evaluated in this article, or claim that may be made by its manufacturer, is not guaranteed or endorsed by the publisher.
